# A long-read draft assembly of the Chinese mantis (Mantodea: Mantidae: *Tenodera sinensis*) genome reveals patterns of ion channel gain and loss across Arthropoda

**DOI:** 10.1093/g3journal/jkae062

**Published:** 2024-03-22

**Authors:** Jay K Goldberg, R Keating Godfrey, Meghan Barrett

**Affiliations:** Department of Ecology and Evolutionary Biology, University of Arizona, 1041 E Lowell St, Tucson, AZ 85741, USA; Department of Crop Genetics, John Innes Centre, Norwich Research Park, Colney Ln, Norwich, Norfolk NR4 7UH, UK; Department of Biological Sciences, Florida International University, 11200 SW 8th St, Miami, FL 33199, USA; Department of Biology, Indiana University Purdue University Indianapolis, 420 University Blvd, Indianapolis, IN 46202, USA

**Keywords:** Chinese mantis, draft genome, nociception, ion channels

## Abstract

Praying mantids (Mantodea: Mantidae) are iconic insects that have captivated biologists for decades, especially the species with cannibalistic copulatory behavior. This behavior has been cited as evidence that insects lack nociceptive capacities and cannot feel pain; however, this behaviorally driven hypothesis has never been rigorously tested at the genetic or functional level. To enable future studies of nociceptive capabilities in mantids, we sequenced and assembled a draft genome of the Chinese praying mantis (*Tenodera sinensis*) and identified multiple classes of nociceptive ion channels by comparison to orthologous gene families in Arthropoda. Our assembly—produced using PacBio HiFi reads—is fragmented (total size = 3.03 Gb; N50 = 1.8 Mb; 4,966 contigs), but is highly complete with respect to gene content (BUSCO complete = 98.7% [odb10_insecta]). The size of our assembly is substantially larger than that of most other insects, but is consistent with the size of other mantid genomes. We found that most families of nociceptive ion channels are present in the *T. sinensis* genome; that they are most closely related to those found in the damp-wood termite (*Zootermopsis nevadensis*); and that some families have expanded in *T. sinensis* while others have contracted relative to nearby lineages. Our findings suggest that mantids are likely to possess nociceptive capabilities and provide a foundation for future experimentation regarding ion channel functions and their consequences for insect behavior.

## Introduction

**Fig. 1.**
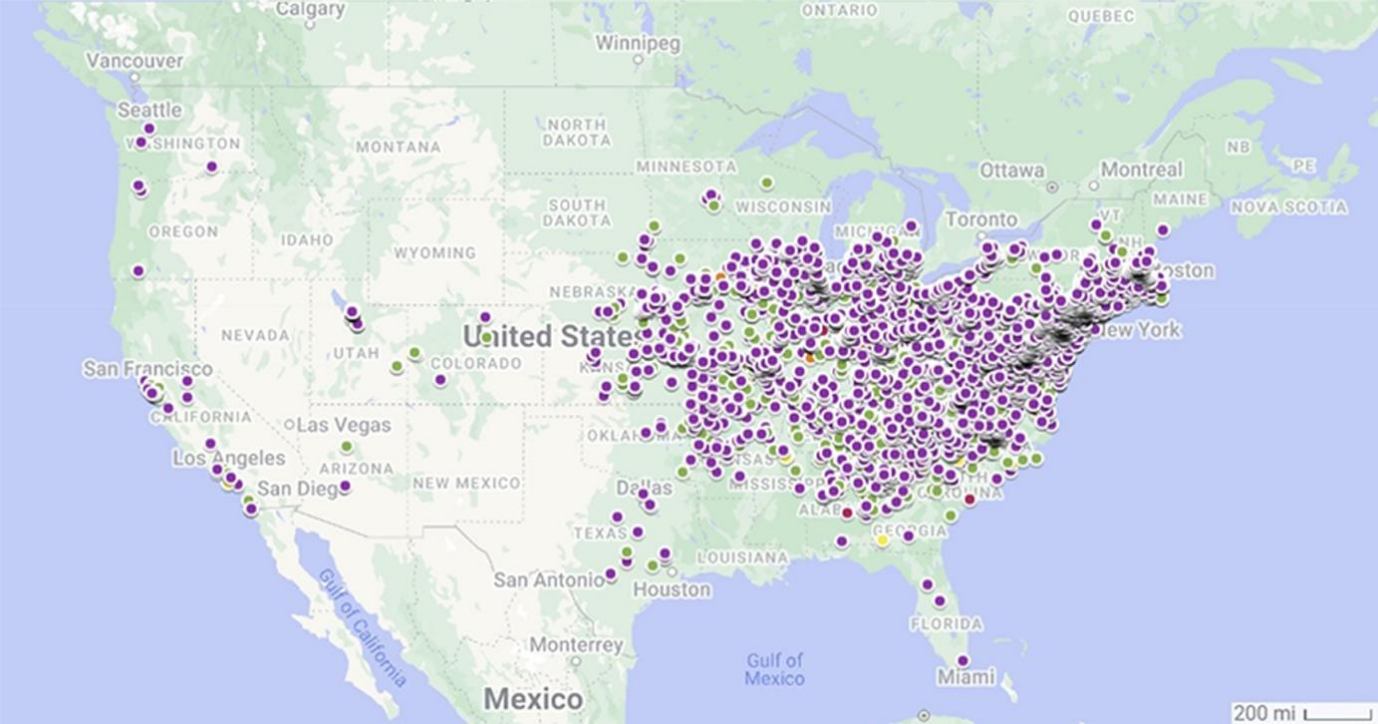
GBIF occurrence records of *T. sinensis* in the United States as of 2023. Color indicates year of record (red, pre-2000; orange, 2000–2009; yellow, 2010–2014; green, 2015–2019; purple, 2020–2023).

The Chinese mantis, *Tenodera sinensis* (Mantidae), is a large-bodied mantid species native to Asia and the nearby islands. In the late 1800s, the species was accidentally introduced to the Philadelphia area (Blatchley 1920) and has become established throughout the contiguous United States (according to data available from the Global Biodiversity Information Facility, GBIF.org 2023; Fig. 1).

As a successful, invasive, generalist predator (Crowder and Snyder 2010), *T. sinensis* has become an important ecological study species in its nonnative range (Moran and Hurd 1994; Moran *et al*. 1996; Fagan *et al*. 2002). *Tenodera sinensis* are ambush predators, spending 93% of their time waiting for prey (Ratchet and Hurd 1983). Depending on life stage and body size (Hurd *et al*. 2015), these mantises eat insects (including other mantises), spiders, slugs, and even hummingbirds (Hurd 1988; Iwasaki 1998; Verchot 2014; Mebs *et al*. 2017; Wilson Rankin *et al*. 2023).

*Tenodera sinensis* is probably most well known as a study system for sexual cannibalism, where females may eat males, before, during, or after copulation, though field observations suggest feeding on the male during copulation is relatively rare (<10% of cases; Hurd *et al*. 1994). Food limitation during oogenesis is expected to drive female mantises to continuously attract mates which, if consumed, increase fecundity (Brown and Barry 2016; O’Hara and Brown 2021). Males may make up 63% of female diets during this crucial reproductive period (Hurd *et al*. 1994). Males are known to adjust their approach behaviors in response to the perceived threat level posed by a female and female encounter rates (Lelito and Brown 2006; Brown *et al*. 2012); unlike some other sexually cannibalistic species, male *T. sinensis* are not self-sacrificial (Buskirk *et al*. 1984).

Despite males’ well-studied risk avoidance behaviors that suggest a lack of complicity (see Lelito and Brown 2006 and discussion in Christensen and Brown 2018), sexual cannibalism in mantids has featured prominently in discussions of the plausibility of insect pain and, thus, sentience (e.g. as in Eisemann *et al*. 1984; though see Gibbons and Sarlak 2020 and Barrett and Fischer 2024). Male mantises may be unable to perceive (minimally) mechanically noxious stimuli, which animals typically perceive using nociceptive ion channels in their peripheral sensory neurons. Eisemann *et al*. (1984) suggest that the lack of nociceptive ability, as demonstrated by males continuing to mate while being cannibalized (e.g. behavioral nonresponsiveness), precludes any plausible further possibility of pain experience. This would make mantids an interesting species for studies of insect pain perception, a field of growing research interest given the development of large-scale insect farming and associated welfare concerns (e.g. van Huis 2021; Gibbons *et al*. 2022; Barrett and Adcock 2023).

Here, we present an assembly of the Chinese praying mantis genome (*T. sinensis*) and combine it with existing genomic resources to study the evolution of nociceptive channel evolution across the arthropod phylum. We find that mantids have genes that encode many well-studied arthropod nociceptors dedicated to perceiving diverse noxious stimuli (mechanical, chemical, and thermal). Across the arthropods, we find that some channel families are conserved, whereas copy numbers vary greatly in others. We discuss the potential factors that may underlie this variation and the implications thereof for rearing and domestication of arthropods.

## Materials and methods

### Mantis rearing

**Fig. 2.**
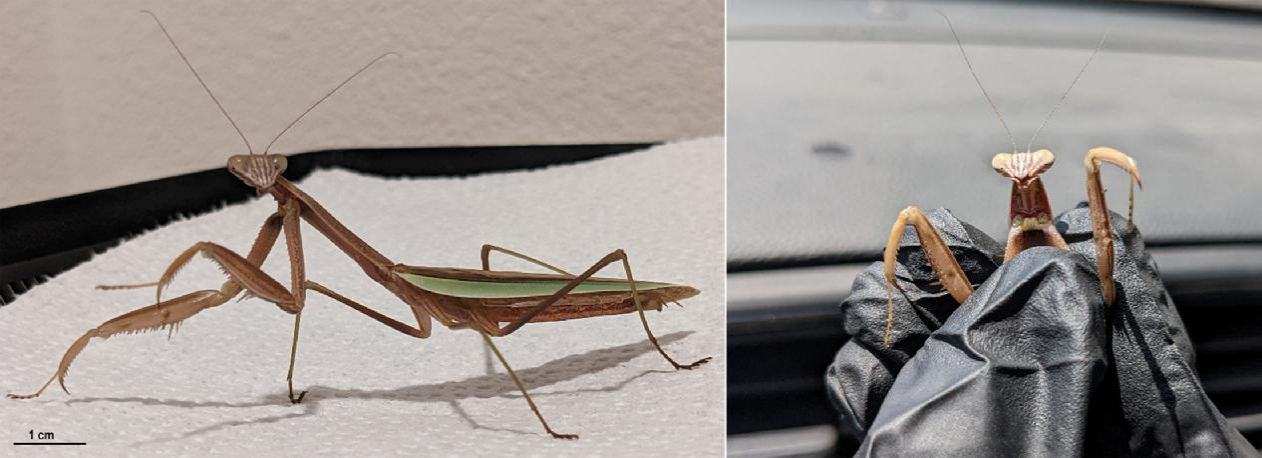
Photos of adult female *T. sinensis* specimen that was used for genome assembly. Scale bar in the left photo represents approximately 1 cm (the mantid stands at an angle and thus changes depth in the photo).

The specimen used for whole-genome sequencing (adult female; Fig. 2) was reared at 50% relative humidity, 27°C, 14:10 L:D from a set of 6 oothecae purchased from Carolina Biological in spring 2023. Mantises were reared collectively until the third instar, then reared in separate containers and fed flightless *Drosophila melanogaster* and *Drosophila hydei*, *Acheta domesticus* cricket nymphs, and *Tenebrio molitor* mealworm larvae. The single female individual used for analyses (Fig. 2) was flash frozen on liquid nitrogen and sent to the Arizona Genomics Institute (AGI, Tucson, AZ, United States) for DNA extraction and sequencing. A female specimen was chosen as they are the homogametic sex in mantises (Paliulis *et al*. 2022).

### DNA extraction

High molecular weight DNA was extracted from ground thoracic tissue in an extraction buffer with Tris HCl buffer 0.1 M, pH 8.0, EDTA 0.1 M, pH 8, SDS 1%, and Proteinase K in 50°C for 60 min. Mixture was spun down, and aqueous phase was transferred to a new tube; 5 M potassium acetate was added, precipitated on ice, and spun down. After centrifugation, the supernatant was gently extracted with 24:1 chloroform:isoamyl alcohol. The upper phase was transferred to a new tube and DNA precipitated with iso-propanol. DNA was collected by centrifugation, washed with 70% ethanol, air dried, and dissolved thoroughly in 1× TE followed by RNAse treatment. DNA purity was measured with NanoDrop, DNA concentration was measured with Qubit HS kit (Invitrogen, Carlsbad CA, United States), and DNA size was validated by Femto Pulse System (Agilent, Santa Clara, CA, United States).

### Genome sequencing

DNA was sheared to an appropriate size range (10–20 kb) using Megaruptor 3 (Diagenode, Denville, NJ, United States) followed by SMRTbell cleanup beads. The sequencing library was constructed following manufacturer’s protocols using SMRTbell Prep kit 3.0. The final library was size selected on a Pippin HT (Sage Science, Beverly, MA, United States) using S1 marker with a 10–25 kb size selection. The recovered final library was quantified with Qubit HS kit (Invitrogen, Carlsbad, CA, United States) and size checked on Femto Pulse System (Agilent, Santa Clara, CA, United States). The final library was prepared for sequencing with PacBio Sequel II Sequencing kit 3.1 for HiFi library, loaded on a single Revio SMRT cells, and sequenced in CCS mode for 24 h.

### Genome assembly and annotation

**Fig. 3.**
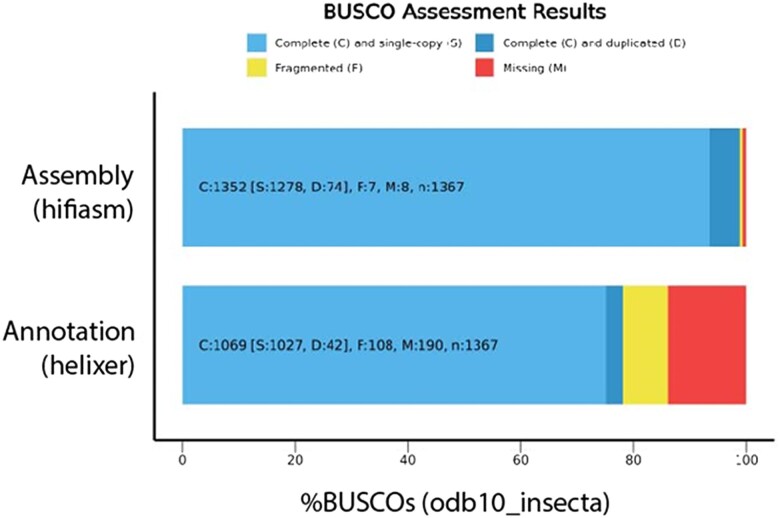
BUSCO assessment of *T. sinensis*: our filtered/polished assembly and its annotation. Annotation assessment was performed in proteome mode.

CCS outputs (i.e. HiFi reads; 6,174,839 reads; 95 Gb Q32; mean length = 15,396) were assembled using hifiasm-0.16.0 (Cheng *et al*. 2021; RRID:SCR_021069) with default settings. Assembly was visualized using Bandage v0.8.1 (Wick *et al*. 2015; RRID:SCR_022772), which also provided contiguity statistics. Contigs assembled from contaminant reads (*N* = 3,832) were identified and filtered from our assembly using the blobtools v1.1 pipeline (Laetsch and Blaxter 2017; RRID:SCR_017618) employing minimap v2-2.24 (Li 2018) for alignment and the nt sequence database for taxonomic identification (Camacho *et al*. 2009). Jellyfish v2.2.10 (Marcais and Kingsford (2011); RRID:SCR_005491) was used for kmer counting (kmer length = 21 bp), and the resulting output was used in the GenomeScope2.0 web portal (Ranallo-Benavidez *et al*. 2020) to estimate genome size (Supplementary Fig. 1). Filtered assembly quality and polishing was carried out using Inspector v1.0.2 (Chen *et al*. 2021; see Supplementary Table 1 for details). Gene content completeness was assessed via BUSCO v5.4.7 (Seppey *et al*. 2019; RRID:SCR_015008) using the Arthropoda_odb10 data set (Fig. 3). Repeat content of the final (filtered and polished) assembly was assessed using RepeatMasker v4.1.3 on default settings (Tarailo-Graovac and Chen 2009; RRID:SCR_012954; Supplementary Table 2) before being structurally annotated with the Helixer v0.3.1 algorithm pipeline (Stiehler *et al*. 2021; Holst *et al*. 2021) using the premade invertebrate training data set. Functional annotation was done using eggnog-mapper v5.1.12 (Huerta-Cepas *et al*. 2019; Cantalapiedra *et al*. 2021; RRID:SCR_002456).

### Gene family analysis

**Fig. 4.**
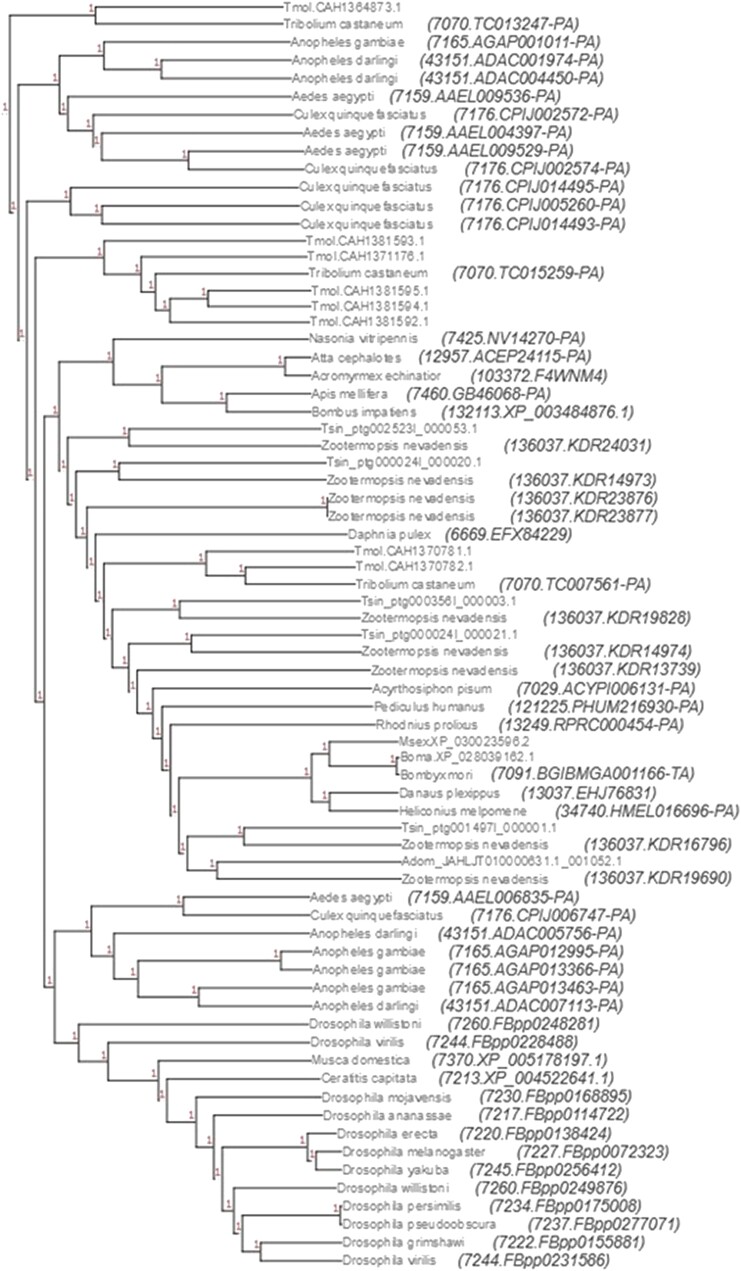
Gene tree showing the relatedness of *painless* genes across our sample of arthropods. *Tenodera sinensis* genes cluster most closely with those of *Z. nevadensis* (Blattodea). Genes with resolved species names were present in the eggnog5 database (Orthofamily 41TMZ), whereas those with only gene IDs at branch tips are those of our additional species (Adom, *A. domesticus*; Boma, *B. mandarina*; Msex, *M. sexta*; Tmol, *T. molitor*; Tsin, *T. sinensis*).

We selected 9 receptor genes associated with nociception when expressed in class III or class IV multidendritic sensory neurons in Arthropoda. This included 5 sensory receptor channels with GO terms for the detection of thermal, mechanical, and/or chemical stimuli involved in the sensory perception of pain (GO0050965, thermal: pain, TRPA1; GO0050966, mechanical: rpk, pain, ppk, ppk26; GO0050968, chemical: ppk, ppk26, TRPA1), TRPm, Pkd2, and NOMPC (ion channels with a role in cold nociception; Turner *et al*. 2016) and 2 mechanoelectrical transduction channels linked to noxious touch sensitization, NOMPC and Piezo (Kim *et al*. 2012; Hehlert *et al*. 2021). These 7 ortholog families were downloaded from the eggnog database (V5.0, http://eggnog5.embl.de). Additional species of interest not present in the database (*Hermetia illucens*, BioProject: PRJEB37575; *T. molitor*, BioProject: PRJNA820846; *Manduca sexta*, BioProject: PRJNA658700; *Penaeus vannamei*, BioProject: PRJNA438564; *A. domesticus*, BioProject: PRJNA706033; *Bombyx mandarina*, BioProject: PRJDB13954; Xiang *et al*. 2018; Zhang *et al*. 2019; Generalovic *et al*. 2021; Gershman *et al*. 2021; Dossey *et al*. 2023; Kaur *et al*. 2023) were manually added to our list by functionally annotating available protein sequences using eggnog-mapper, which uses the eggnog5.0 database to identify orthologous gene families. *Acheta domesticus* did not have publicly available protein sequences; thus, we structurally annotated it using Helixer v0.3.1 (Stiehler *et al*. 2021; Holst *et al*. 2021) before functional annotation. Phylogenetic analysis (sequence alignment and tree building) of PAINLESS peptide sequences (Fig. 4) was done using Clustal Omega (https://www.ebi.ac.uk/Tools/msa/clustalo/; Madeira *et al*. 2022; RRID:SCR_001591) before visualization using the ETE TreeView web portal (http://etetoolkit.org/treeview/; Huerta-Cepas *et al*. 2016). Gene families of interest were also obtained from the newest (v6; Supplementary Table 3) eggnog database (eggnog6.embl.de; Hernández-Plaza *et al*. 2023) but additional species were not added.

## Results and discussion

### *Tenodera sinensis* genome assembly and annotation

The initial assembly was highly fragmented by long-read genome assembly standards (number of contigs = 8,798; N50 = 1.6 Mb; total size = 3.3 Gb) and roughly 20% larger than our GenomeScope prediction (2.7 Gb; Supplementary Fig. 1). Blobtools analysis determined that the assembly was heavily contaminated with viral and bacterial contigs (*N* = 3,832, Supplementary Fig. 2; 18.81% of raw reads, Supplementary Table 1). Our contaminant-filtered assembly was still found to be fragmented and larger than expected (number of contigs = 4,966; N50 = 1.8 Gb; total length = 3.03 Gb; Supplementary Table 1). This is similar to genome sizes reported for other mantis species (*Hymenopus coronatus* = 2.88 Gb; *Deroplatys lobata* = 3.96 Gb; Huang *et al*. 2023). RepeatMasker found that 68.83% of the genome was composed of repetitive elements (Supplementary Table 2), predominantly retroelements (18.41%) and DNA transposons (18.08%). Inspector analysis found that our assembly had a number of structural errors (*N* = 593) and small-scale errors (*N* = 83,917; 27.7 per Mb), but polishing was able to remove most small-scale errors (after polishing *N* = 8,788; 2.9 per Mb; see Supplementary Table 1 for details). Despite assembly errors, BUSCO analysis determined that our assembly was highly complete [odb10_insecta complete = 98.9% (single copy = 93.5%, duplicated = 5.4%), fragmented = 0.5%, missing = 0.6%; Fig. 3]. Helixer structurally annotated 24,673 genes in our assembly, 14,092 of which were functionally annotated via comparison to the eggnog-mapper database using only orthology to Arthropoda. BUSCO assessment of our annotation showed that a substantial portion of total gene content is likely not represented [odb10_insecta complete = 78.2% (single copy = 75.1%, duplicated = 3.1%), fragmented = 7.9%, missing = 13.9%; Fig. 3]; however, this was also the case (albeit less drastically) for our Helixer annotation of *A. domesticus* [odb10_insecta complete = 86.7% (single copy = 82.7%, duplicated = 4.0%), fragmented = 7.4%, missing = 5.9%; BUSCO complete in assembly = 94.8%; Dossey *et al.* 2023] suggesting there may be biases in Helixer's premade invertebrate training data set, which is dominated by insects in the orders Diptera and Lepidoptera (details regarding Helixer training data sets can be found at https://uni-duesseldorf.sciebo.de/s/lQTB7HYISW71Wi0). Thus, the lower than expected recovery of single-copy orthologs may represent this bias rather than the quality of our assembly. Summary of assembly and annotation statistics is found in Table 1, with further details provided in Supplementary Table 1.

**Table 1.** Summary statistics of the *T. sinensis* draft assembly and annotation.

Feature	Value
Assembly size	3,032,411,605 bp
Number of contigs	4,966
Largest contig	16,238,500 bp
Contig N50	1,806,165 bp
Repeat content	68.83%
GC content	37.99%
Assembly BUSCO complete (odb10_insecta)	98.90%
Annotation BUSCO complete (odb10_insecta)	78.20%
Number of genes	24,673
Functionally annotated genes	14,092

### Variation in ion channel copy numbers in *T. sinensis*

*Tenodera sinensis* was found to have at least 1 copy of 8 of 9 genes for receptor channels of interest, with the exception being *pkd2* (Table 2). Pkd2 acts as a direct cold sensor in class III md neurons in *D. melanogaster* and is necessary (alongside NOMPC and TRPm) for aversive responses to cold behavior (Turner *et al*. 2016). *Tenodera sinensis* was found to have an expansion in both *nompC* and *trpm* copy number (from 1 each in *D. melanogaster* to 3 each in *T. sinensis*). The lack of *pkd2* could suggest an inability to directly sense aversive levels of cold in *T. sinensis* despite the presence of *nompC* and *trpm*, but given its function has been characterized solely in *D. melanogaster* and we could not detect an ortholog in many insect genomes, it is difficult to infer this with confidence. Further study of *T. sinensis* behavior and physiology would be needed to assess function. Other orders of insects have evolved novel thermal nociceptors following the loss of other thermosensitive nociceptive ion channels (e.g. duplication and neofunctionalization of waterwitch into HsTRPA to complement the loss of TRPA1 in the Hymenoptera; Kohno *et al*. 2010). Further, there is often more than 1 receptor for assessing potentially noxious thermal information (e.g. the heat sensitivity of pyrexia, painless, and TRPA1; Tracey *et al*. 2003; Lee *et al*. 2005 ; Wang *et al*. 2009).

**Table 2.** Ion channel gene families obtained from the eggnog5 database.

		Orthogroup	41TMZ	41U79	41UGV	41UWY	41VXR	41X6F	41XIN
Order	Family	Representative gene name	*pain*	*NompC*	*trpm*	*Piezo*	*Pkd2*	*ppk/rpk/ppk26*	*TrpA1*
Trombidiformes	Tetranychidae	*Tetranychus urticae*	0	2	1	2	1	0	0
Chilopoda	Geophilomorpha	*Strigamia maritima*	0	1	2	2	1	0	2
Anomopoda	Daphniidae	*Daphnia pulex*	1	2	2	0	0	0	0
Decapoda	Penaeidae	*Penaeus vannamei*	0	1	2	2	0	0	2
Blattodea	Archotermopsidae	*Zootermopsis nevadensis*	9	0	1	1	6	0	1
Mantodea	Mantidae	*Tenodera sinensis*	5	3	3	2	0	5	1
Psocodea	Pediculidae	*Pediculus humanus*	1	1	1	3	1	0	1
Hemiptera	Aphididae	*Acyrthosiphon pisum*	1	1	2	3	1	3	1
Hemiptera	Reduviidae	*Rhodnius prolixus*	1	1	2	1	1	1	1
Orthoptera	Gryllidae	*Acheta domesticus*	1	4	1	2	0	12	1
Hymenoptera	Formicidae	*Acromyrmex echinatior*	1	4	1	1	0	0	0
Hymenoptera	Formicidae	*Atta cephalotes*	1	5	2	1	0	0	0
Hymenoptera	Braconidae	*Microplitis demolitor*	0	2	1	2	0	0	0
Hymenoptera	Pteromalidae	*Nasonia vitripennis*	1	1	1	2	0	0	0
Hymenoptera	Apidae	*Apis mellifera*	1	4	1	1	0	0	0
Hymenoptera	Apidae	*Bombus impatiens*	1	2	1	1	0	0	0
Coleoptera	Tenebrionidae	*Tribolium castaneum*	3	1	1	1	1	17	1
Coleoptera	Tenebrionidae	*Tenebrio molitor*	8	1	1	1	1	19	1
Lepidoptera	Nymphalidae	*Danaus plexippus*	1	1	1	1	0	3	1
Lepidoptera	Nymphalidae	*Heliconius melpomene*	1	1	2	1	0	1	1
Lepidoptera	Sphingidae	*Manduca sexta*	1	2	8	7	0	4	2
Lepidoptera	Bombycidae	*Bombyx mandarina*	1	2	5	8	0	3	1
Lepidoptera	Bombycidae	*Bombyx mori*	1	1	2	2	0	2	1
Diptera	Culicidae	*Aedes aegypti*	4	2	2	1	0	20	2
Diptera	Culicidae	*Anopheles darlingi*	4	1	1	1	0	4	2
Diptera	Culicidae	*Anopheles gambiae*	4	1	1	1	0	6	1
Diptera	Culicidae	*Culex quinquefasciatus*	6	2	2	3	0	31	2
Diptera	Tephritidae	*Ceratitis capitata*	1	1	1	1	2	3	1
Diptera	Drosophilidae	*Drosophila ananassae*	1	2	2	1	1	3	1
Diptera	Drosophilidae	*Drosophila erecta*	1	1	2	1	1	3	1
Diptera	Drosophilidae	*Drosophila grimshawi*	1	1	2	2	3	3	1
Diptera	Drosophilidae	*Drosophila melanogaster*	1	1	1	5	1	3	1
Diptera	Drosophilidae	*Drosophila mojavensis*	1	1	2	2	4	4	1
Diptera	Drosophilidae	*Drosophila persimilis*	1	1	2	1	5	4	2
Diptera	Drosophilidae	*Drosophila pseudoobscura*	1	1	2	2	5	3	2
Diptera	Drosophilidae	*Drosophila virilis*	2	1	2	2	3	3	1
Diptera	Drosophilidae	*Drosophila willistoni*	2	1	1	2	1	3	1
Diptera	Drosophilidae	*Drosophila yakuba*	1	1	2	1	1	3	1
Diptera	Muscidae	*Musca domestica*	1	1	1	2	1	11	2
Diptera	Hermetiinae	*Hermetia illucens*	0	2	2	1	0	2	6

This table includes gene counts of species included in the database and those obtained from eggnog-mapper outputs of additional species.

Prior arguments by Eisemann *et al*. (1984) and others have hypothesized that behavioral nonresponsiveness to injury in insects, such as an *T. sinensis* sexual cannibalism, suggests insects are unlikely to perceive (minimally, mechanically) noxious stimuli and thus unlikely to have an adaptive role for pain and sentience. However, since these publications, the genetic components of nociception have been found to be ancient and highly conserved (e.g. Peng *et al*. 2015); it is, therefore, unsurprising that our data suggest that chemical, mechanical, and thermal nociception are plausible in *T. sinensis*. Still, further tests that confirm (1) these channels are expressed in multidendritic sensory neurons and (2) they have a nociceptive function in the Mantodea will be important for conclusively demonstrating nociception in mantids.

Differential expression and splicing into novel isoforms can lead to different functional roles of these receptors in different cell types (e.g. *dTRPA1*; Zhong *et al*. 2012; Gu *et al*. 2022). Amino acid substitution can also lead to variation in channel sensitivity to noxious stimuli, even for orthologous channels (e.g. loss of chemical sensitivity in *Si*HsTRPA vs *Am*HsTRP; Wang *et al*. 2018). Altogether, these various mechanisms for generating functional plasticity of these receptors in different tissue types suggest moderate caution should be employed when suggesting the presence or absence of a specific gene corresponds to the presence or absence of a specific nociceptive ability in Mantodea. Still, we may broadly expect some perception of noxious stimuli to be highly plausible given this genetic information and the ancient and highly conserved nature of many of these genes. If male mantises are, indeed, able to perceive a mechanically noxious stimulus (such as female cannibalism), this suggests mechanisms other than a lack of nociceptive perception underpin any instances of apparent behavioral unresponsiveness (see Gibbons and Sarlak 2020). Instead, our genetic data support prior behavioral evidence that shows male mantises attempt to avoid being cannibalized (Lelito and Brown 2006; Brown *et al*. 2012) and provides further support for the ancient and highly conserved nature of the peripheral components of pain pathways across Arthropoda.

### Variation in ion channel copy number across Arthropoda

All the gene families except the *ppk/rpk/ppk26* family saw reasonably similar ranges of copy number across the surveyed arthropods (from 0 up to 6–9 copies). However, greater variation (0–31 copies) was found in the *ppk/rpk/ppk26* family, with roles in mechanical and chemical nociception. This included substantial intraorder variation (e.g. 2 copies in *H. illucens*, 11 copies in *Musca domestica*, and 31 copies in *Culex quinquefasciatus*, all dipterans). Prior research on mosquitoes has found that additional ion channel gene copies play a role in nonnociceptive sensory processes, specifically heat perception involved with prey location (Wang *et al*. 2009). Our results suggest that ion channel copy numbers are highly variable and that they are likely to play an important role in the evolution of Arthropod sensory systems, but more precise studies of expression dynamics and cellular functions are necessary to better understand this process.

Our search also specifically focused on insects that are mass reared (such as the black soldier fly, *H. illucens*) or even domesticated (such as the silkworm moth, *Bombyx mori*) to see if there were any patterns of ion channel loss or duplication associated with the novel ecological conditions posed by rearing at scale on farms under significant human management. Generally, farmed species retained at least 1 copy of all genes except *pkd2*, with only *T. molitor* and *M. domestica* having a copy of this gene. This could suggest, as in *T. sinensis*, some loss of cold nociception capabilities that should be confirmed functionally (and considered alongside the aforementioned caveats).

The only other gene loss event was *painless* in *H. illucens*; however, *H. illucens* had significant duplication of *trpa1* (6 copies), where no other arthropod species in our study had more than 2 copies of this gene. It is possible the additional copies of *trpa1* may serve to recover some of the lost functions of *painless* as both are TRPA proteins that have roles, for instance, in thermal nociception in other Diptera (Neely *et al*. 2011; Hwang *et al*. 2012). *Tenebrio molitor* had significant duplication of *painless* (8 copies) and *ppk/rpk/ppk26* (19 copies). *Acheta domesticus* also had significant duplication of *ppk/rpk/ppk26* (12 copies). *Bombyx*  *mandarina* had significant duplication of *piezo* (8 copies) and *trpm* (5 copies). These genes were both highly expressed in the 2 lepidopterans—*B.*  *mandarina* and *Manduca*  *sexta—*but not in any other order we reviewed. Overall, these results suggest that domestication and/or mass rearing do not result in nociceptive ion channel loss in insects, which means it will still be important to monitor and reduce the occurrence of noxious stimuli in mass-rearing facilities as part of ethical mini-livestock husbandry and slaughter.

## Conclusion

In summary, we have produced a high-quality draft genome of the Chinese mantis (*T. sinensis*) and used it to identify multiple putative nociceptive ion channels. Comparative analyses suggest that some of these genes have undergone multiple duplications that are shared with Blattodea, whereas others have been lost in these closely related clades. These findings suggest that mantids are likely capable of perceiving different types of noxious stimuli (extreme heat/cold, mechanical damage, etc.). Future research into their precise functional roles will elucidate the evolution of nociception in an iconic sexually cannibalistic species.

## Supplementary Material

jkae062_Supplementary_Data

## Data Availability

A male nymph and adult female from the same set of oothecae were vouchered at Purdue Entomological Research Collection (PERC 0154978 and PERC 0154979). The entire individual used for sequencing was destroyed during sampling. Raw reads and our assembly are available under BioProject PRJNA1036567. No new code was written for this study, but scripts used to run existing programs are found at https://github.com/caterpillar-coevolution/Tsinensis_genome_V1 alongside annotation files. Supplemental material available at G3 online.
