# Supplementary material for: A long-read draft assembly of the Chinese mantis (Mantodea: Mantidae: Tenodera sinensis) genome reveals patterns of ion channel gain and loss across Arthropoda
Source: G3 (Bethesda). 2024 Mar 22;14(6):jkae062. doi: 10.1093/g3journal/jkae062 (PMC11152070; doi:10.1093/g3journal/jkae062)
Supplement: jkae062_Supplementary_Data [file jkae062_supplementary_data.docx]

**Supplementary Materials**

**
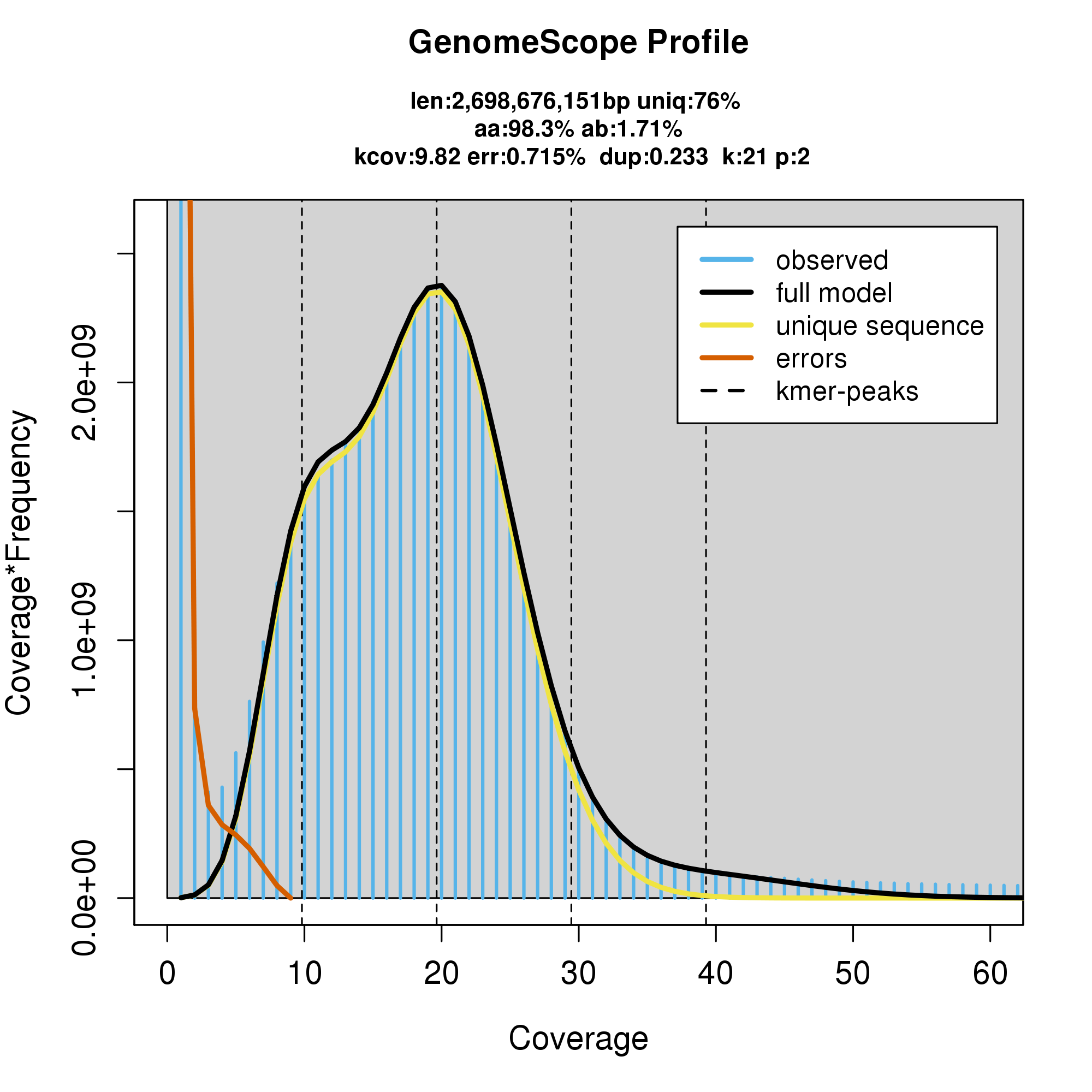
**

**Figure S1.** Genomescope profile plot showing the results of kmer coverage analysis performed on our raw sequencing reads.

**
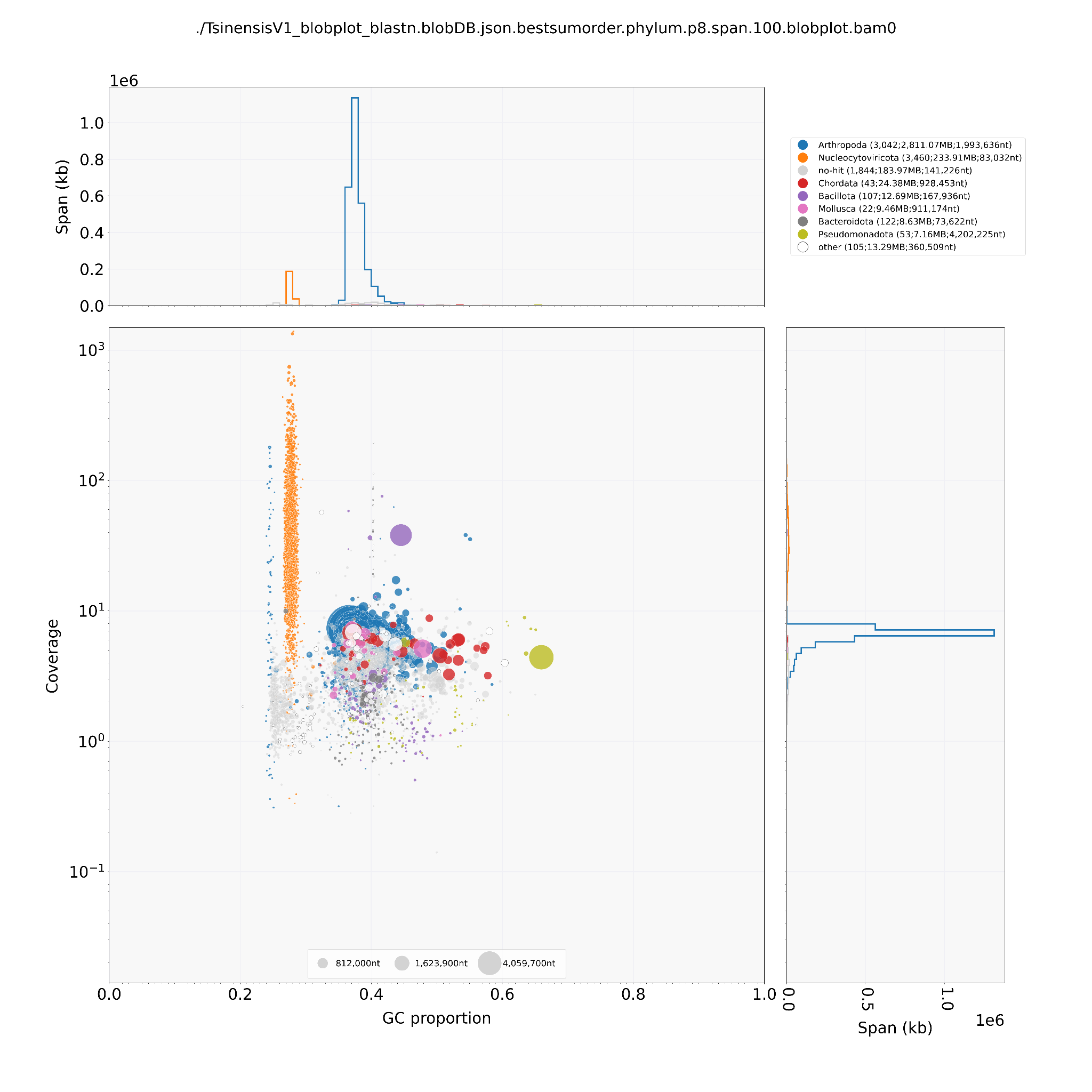
**

**Figure S2.** Blobplot showing the presence of substantial viral and bacterial contamination in our genome assembly. Contigs identified as viral or bacterial (N = 3832) were filtered out of our assembly.

**Table S1.** Summary statistics of genome assembly before and after polishing with Inspector

|  | **Before** | **After** |
| --- | --- | --- |
| Number of contigs | 4966 | 4966 |
| Number of contigs > 10000 bp | 4966 | 4966 |
| Number of contigs >1000000 bp | 837 | 837 |
| Total length | 3032411541 | 3032411605 |
| Total length of contigs > 10000 bp | 3032411541 | 3032411605 |
| Total length of contigs >1000000bp | 2084261305 | 2084260901 |
| Longest contig | 16238473 | 16238500 |
| Second longest contig length | 14940332 | 14940332 |
| N50 | 1806180 | 1806165 |
| N50 of contigs >1Mbp | 1806180 | 1806165 |
| Mapping rate /% | 81.19 | 81.19 |
| Split-read rate /% | 7.83 | 7.83 |
| Depth | 25.541 | 25.5411 |
| Mapping rate in large contigs /% | 61.28 | 61.28 |
| Split-read rate in large contigs /% | 6.64 | 6.63 |
| Depth in large conigs | 28.1542 | 28.1532 |
| Structural error | 593 | 606 |
| Expansion | 386 | 391 |
| Collapse | 172 | 177 |
| Haplotype switch | 31 | 34 |
| Inversion | 4 | 4 |
| Small-scale assembly error /per Mbp | 27.67335464 | 2.898023469 |
| Total small-scale assembly error | 83917 | 8788 |
| Base substitution | 61937 | 7186 |
| Small-scale expansion | 10715 | 703 |
| Small-scale collapse | 11265 | 899 |
| QV | 38.4297348 | 39.15840409 |

**Table S2.** Summary of GC content and repetitive element content from RepeatMasker

| **Repeat Type** | **Number of elements** | **Bases occupied** | **Percentage of genome** |
| --- | --- | --- | --- |
| Total Retroelements | 1187304 | 558139741 bp | 18.41% |
| SINEs | 254592 | 51934143 bp | 1.71% |
| Penelope | 200995 | 53852202 bp | 1.78% |
| LINEs | 821784 | 414942122 bp | 13.68% |
| CRE/SLACS | 281 | 58648 bp | 0.00% |
| L2/CR1/Rex | 75067 | 29816073 bp | 0.98% |
| R1/LOA/Jockey | 138271 | 104118566 bp | 3.43% |
| R2/R4/NeSL | 8908 | 4107497 bp | 0.14% |
| RTE/Bov-B | 131559 | 49351272 bp | 1.63% |
| L1/CIN4 | 0 | 0 bp | 0.00% |
| LTR elements | 110928 | 91263476 bp | 3.01% |
| BEL/Pao | 12938 | 6607057 bp | 0.22% |
| Ty1/Copia | 1349 | 1179916 bp | 0.04% |
| Gypsy/DIRS1 | 58027 | 71662015 bp | 2.36% |
| Retroviral | 0 | 0 bp | 0.00% |
| DNA transposons | 1648380 | 548224816 bp | 18.08% |
| hobo-Activator | 287683 | 103164071 bp | 3.40% |
| Tc1-IS630-Pogo | 1076091 | 344081300 bp | 11.35% |
| En-Spm | 0 | 0 bp | 0.00% |
| MuDR-IS905 | 0 | 0 bp | 0.00% |
| PiggyBac | 35499 | 19370727 bp | 0.64% |
| Tourist/Harbinger | 12531 | 2267210 bp | 0.07% |
| Other (Mirage, P-element, Transib) | 28674 | 7487191 bp | 0.25% |
| Rolling-circles | 332089 | 65331100 bp | 2.15% |
| Unclassified | 3101589 | 753447777 bp | 24.85% |
| Total interspersed repeats | N/A | 1859812334 bp | 61.33% |
| Small RNA | 100248 | 14964612 bp | 0.49% |
| Satellites | 12858 | 777254 bp | 0.03% |
| Simple repeats | 676621 | 140896021 bp | 4.65% |
| Low complexity | 39761 | 5278509 bp | 0.17% |

**Table S3.** Ion channel gene families obtained from the EggNog6 database. Only genes present within the database are included.

| Name | taxID | HI4MB_painless | HI5CD_PKD2 | HI79A_NompC | HIMUV_PIEZO | HIU8H_TRPA1 | HIWMP_TRMP3 | HIXRG_PPK26 |
| --- | --- | --- | --- | --- | --- | --- | --- | --- |
| *Acromyrmex echinatior* | 103372 | 1 | 0 | 2 | 1 | 1 | 1 | 1 |
| *Acyrthosiphon pisum* | 7029 | 1 | 2 | 1 | 3 | 3 | 2 | 6 |
| *Aedes aegypti* | 7159 | 4 | 0 | 9 | 1 | 1 | 4 | 18 |
| *Aedes albopictus* | 7160 | 5 | 0 | 13 | 5 | 2 | 1 | 21 |
| *Agrilus planipennis* | 224129 | 2 | 0 | 0 | 0 | 3 | 0 | 3 |
| *Anopheles albimanus* | 7167 | 7 | 0 | 3 | 1 | 3 | 1 | 6 |
| *Anopheles atroparvus* | 41427 | 4 | 0 | 3 | 1 | 3 | 1 | 6 |
| *Anopheles christyi* | 43041 | 4 | 0 | 3 | 1 | 3 | 2 | 5 |
| *Anopheles coluzzii* | 1518534 | 4 | 0 | 6 | 1 | 3 | 1 | 8 |
| *Anopheles culicifacies* | 139723 | 2 | 0 | 4 | 1 | 3 | 4 | 8 |
| *Anopheles darlingi* | 43151 | 3 | 0 | 4 | 1 | 2 | 2 | 4 |
| *Anopheles dirus* | 7168 | 6 | 0 | 6 | 1 | 4 | 3 | 9 |
| *Anopheles epiroticus* | 199890 | 3 | 0 | 4 | 1 | 3 | 1 | 7 |
| *Anopheles farauti* | 69004 | 3 | 0 | 4 | 1 | 3 | 1 | 7 |
| *Anopheles funestus* | 62324 | 6 | 0 | 4 | 1 | 3 | 3 | 7 |
| *Anopheles gambiae* | 7165 | 6 | 0 | 3 | 1 | 3 | 1 | 7 |
| *Anopheles melas* | 34690 | 2 | 0 | 5 | 1 | 5 | 2 | 7 |
| *Anopheles merus* | 30066 | 6 | 0 | 5 | 1 | 2 | 0 | 7 |
| *Anopheles minimus* | 112268 | 4 | 0 | 6 | 1 | 3 | 1 | 7 |
| *Anopheles quadriannulatus* | 34691 | 6 | 0 | 4 | 1 | 3 | 2 | 8 |
| *Anopheles sinensis* | 74873 | 23 | 0 | 10 | 1 | 2 | 1 | 8 |
| *Anopheles stephensi* | 30069 | 2 | 0 | 4 | 1 | 3 | 1 | 10 |
| *Apis cerana cerana* | 94128 | 1 | 0 | 2 | 1 | 2 | 1 | 1 |
| *Apis mellifera* | 7460 | 1 | 0 | 3 | 1 | 2 | 1 | 1 |
| *Armadillidium vulgare* | 13347 | 2 | 1 | 1 | 1 | 0 | 1 | 0 |
| *Asbolus verrucosus* | 1661398 | 2 | 2 | 15 | 2 | 2 | 1 | 31 |
| *Atta cephalotes* | 12957 | 1 | 0 | 2 | 1 | 1 | 1 | 1 |
| *Atta colombica* | 520822 | 1 | 0 | 0 | 1 | 0 | 1 | 1 |
| *Blattella germanica* | 6973 | 14 | 1 | 27 | 1 | 2 | 1 | 12 |
| *Bombyx mori* | 7091 | 1 | 0 | 1 | 2 | 3 | 2 | 3 |
| *Camponotus floridanus* | 104421 | 1 | 0 | 1 | 1 | 15 | 1 | 1 |
| *Chilo suppressalis* | 168631 | 0 | 0 | 1 | 1 | 1 | 1 | 3 |
| *Clunio marinus* | 568069 | 6 | 0 | 2 | 3 | 2 | 1 | 5 |
| *Cryptotermes secundus* | 105785 | 6 | 0 | 10 | 1 | 2 | 5 | 3 |
| *Culex quinquefasciatus* | 7176 | 6 | 0 | 12 | 3 | 2 | 3 | 26 |
| *Cyphomyrmex costatus* | 456900 | 1 | 0 | 1 | 1 | 0 | 1 | 0 |
| *Danaus plexippus plexippus* | 278856 | 1 | 0 | 2 | 0 | 3 | 3 | 3 |
| *Daphnia magna* | 35525 | 1 | 0 | 5 | 0 | 0 | 2 | 4 |
| *Daphnia pulex* | 6669 | 1 | 0 | 8 | 0 | 0 | 2 | 6 |
| *Dendroctonus ponderosae* | 77166 | 1 | 1 | 3 | 2 | 8 | 1 | 5 |
| *Diaphorina citri* | 121845 | 0 | 2 | 7 | 11 | 1 | 4 | 2 |
| *Dinothrombium tinctorium* | 1965070 | 0 | 2 | 2 | 1 | 0 | 3 | 0 |
| *Drosophila ananassae* | 7217 | 1 | 1 | 4 | 3 | 2 | 1 | 6 |
| *Drosophila busckii* | 30019 | 2 | 2 | 1 | 1 | 2 | 0 | 5 |
| *Drosophila grimshawi* | 7222 | 1 | 3 | 1 | 2 | 2 | 2 | 5 |
| *Drosophila guanche* | 7266 | 1 | 6 | 2 | 3 | 2 | 1 | 5 |
| *Drosophila melanogaster* | 7227 | 1 | 1 | 1 | 2 | 2 | 1 | 5 |
| *Drosophila navojoa* | 7232 | 1 | 4 | 1 | 3 | 2 | 1 | 5 |
| *Drosophila persimilis* | 7234 | 1 | 5 | 1 | 1 | 2 | 2 | 7 |
| *Drosophila pseudoobscura pseudoobscura* | 46245 | 3 | 5 | 1 | 4 | 2 | 1 | 5 |
| *Drosophila sechellia* | 7238 | 1 | 1 | 1 | 1 | 2 | 2 | 5 |
| *Drosophila simulans* | 7240 | 1 | 1 | 1 | 1 | 2 | 2 | 4 |
| *Drosophila virilis* | 7244 | 2 | 4 | 1 | 4 | 2 | 1 | 5 |
| *Drosophila willistoni* | 7260 | 1 | 1 | 1 | 4 | 2 | 1 | 5 |
| *Dufourea novaeangliae* | 178035 | 1 | 0 | 2 | 0 | 2 | 1 | 1 |
| *Eumeta japonica* | 151549 | 1 | 0 | 6 | 2 | 4 | 7 | 10 |
| *Folsomia candida* | 158441 | 0 | 1 | 1 | 1 | 2 | 1 | 4 |
| *Glossina austeni* | 7395 | 0 | 1 | 3 | 1 | 2 | 2 | 5 |
| *Glossina brevipalpis* | 37001 | 1 | 1 | 1 | 1 | 2 | 3 | 2 |
| *Glossina fuscipes fuscipes* | 201502 | 1 | 1 | 0 | 1 | 2 | 2 | 4 |
| *Glossina morsitans morsitans* | 37546 | 1 | 1 | 1 | 2 | 2 | 2 | 6 |
| *Glossina pallidipes* | 7398 | 2 | 1 | 2 | 1 | 2 | 4 | 5 |
| *Glossina palpalis gambiensis* | 67801 | 1 | 1 | 1 | 1 | 2 | 2 | 4 |
| *Habropoda laboriosa* | 597456 | 1 | 0 | 3 | 1 | 3 | 1 | 1 |
| *Harpegnathos saltator* | 610380 | 1 | 0 | 2 | 1 | 1 | 1 | 2 |
| *Heliothis virescens* | 7102 | 1 | 0 | 3 | 1 | 3 | 2 | 2 |
| *Ixodes scapularis* | 6945 | 0 | 1 | 1 | 3 | 0 | 5 | 0 |
| *Laodelphax striatellus* | 195883 | 1 | 1 | 6 | 2 | 2 | 1 | 6 |
| *Lasius niger* | 67767 | 1 | 0 | 2 | 2 | 3 | 3 | 0 |
| *Leptotrombidium deliense* | 299467 | 0 | 2 | 2 | 3 | 0 | 3 | 0 |
| *Lucilia cuprina* | 7375 | 2 | 1 | 1 | 1 | 2 | 0 | 4 |
| *Megaselia scalaris* | 36166 | 0 | 1 | 2 | 1 | 1 | 3 | 8 |
| *Melipona quadrifasciata* | 166423 | 1 | 0 | 2 | 1 | 1 | 1 | 1 |
| *Musca domestica* | 7370 | 1 | 1 | 1 | 3 | 2 | 1 | 7 |
| *Nasonia vitripennis* | 7425 | 1 | 0 | 13 | 1 | 2 | 1 | 1 |
| *Ooceraea biroi* | 2015173 | 1 | 0 | 5 | 1 | 2 | 1 | 1 |
| *Operophtera brumata* | 104452 | 1 | 0 | 2 | 3 | 4 | 5 | 3 |
| *Orchesella cincta* | 48709 | 0 | 1 | 7 | 2 | 5 | 2 | 3 |
| *Papilio machaon* | 76193 | 1 | 0 | 1 | 1 | 3 | 1 | 3 |
| *Papilio xuthus* | 66420 | 1 | 0 | 2 | 1 | 3 | 1 | 3 |
| *Pediculus humanus corporis* | 121224 | 1 | 1 | 1 | 3 | 2 | 1 | 0 |
| *Penaeus vannamei* | 6689 | 1 | 1 | 10 | 2 | 0 | 4 | 0 |
| *Rhodnius prolixus* | 13249 | 1 | 2 | 3 | 1 | 1 | 2 | 2 |
| *Stegodyphus mimosarum* | 407821 | 0 | 4 | 1 | 3 | 0 | 6 | 0 |
| *Stomoxys calcitrans* | 35570 | 5 | 1 | 1 | 1 | 2 | 1 | 7 |
| *Strigamia maritima* | 126957 | 0 | 1 | 1 | 2 | 1 | 2 | 0 |
| *Temnothorax longispinosus* | 300112 | 12 | 0 | 4 | 1 | 0 | 1 | 1 |
| *Tetranychus urticae* | 32264 | 0 | 1 | 3 | 2 | 0 | 1 | 0 |
| *Tigriopus californicus* | 6832 | 8 | 4 | 3 | 0 | 0 | 1 | 9 |
| *Trachymyrmex cornetzi* | 471704 | 1 | 0 | 1 | 1 | 0 | 1 | 1 |
| *Trachymyrmex septentrionalis* | 34720 | 1 | 0 | 2 | 1 | 0 | 1 | 1 |
| *Trachymyrmex zeteki* | 64791 | 1 | 0 | 1 | 1 | 2 | 1 | 1 |
| *Tribolium castaneum* | 7070 | 2 | 1 | 6 | 2 | 1 | 1 | 10 |
| *Trichomalopsis sarcophagae* | 543379 | 1 | 0 | 16 | 1 | 0 | 1 | 1 |
| *Tropilaelaps mercedesae* | 418985 | 0 | 1 | 1 | 2 | 0 | 0 | 0 |
| *Zootermopsis nevadensis* | 136037 | 8 | 7 | 7 | 2 | 4 | 1 | 2 |
